# Incorporating a situational judgement test in residency selections: clinical, educational and organizational outcomes

**DOI:** 10.1186/s12909-024-05310-8

**Published:** 2024-03-26

**Authors:** Anurag Saxena, Loni Desanghere, Kelly Dore, Harold Reiter

**Affiliations:** 1 College of Medicine, University of Saskatchewan, Room 3A10, Health Sciences Bldg., 107 Wiggins Road, Saskatoon, SK S7N 5E5 Canada; 2 College of Medicine, University of Saskatchewan, Saskatoon, Canada; 3 Department of Medicine, McMaster University, Hamilton, Canada, Science and Innovation at Acuity Insights, Toronto, ON Canada; 4 Department of Oncology, McMaster University, Hamilton, ON Canada

**Keywords:** Situational judgement test, Casper, Professionalism, Resident selection, Learner outcomes, Organizational outcomes

## Abstract

**Background:**

Computer-based assessment for sampling personal characteristics (Casper), an online situational judgement test, is a broad measure of personal and professional qualities. We examined the impact of Casper in the residency selection process on professionalism concerns, learning interventions and resource utilization at an institution.

**Methods:**

In 2022, admissions data and information in the files of residents in difficulty (over three years pre- and post- Casper implementation) was used to determine the number of residents in difficulty, CanMEDS roles requiring a learning intervention, types of learning interventions (informal learning plans vs. formal remediation or probation), and impact on the utilization of institutional resource (costs and time). Professionalism concerns were mapped to the 4I domains of a professionalism framework, and their severity was considered in mild, moderate, and major categories. Descriptive statistics and between group comparisons were used for quantitative data.

**Results:**

In the pre- and post- Casper cohorts the number of residents in difficulty (16 vs. 15) and the number of learning interventions (18 vs. 16) were similar. Professionalism concerns as an outcome measure decreased by 35% from 12/16 to 6/15 (*p* < 0.05), were reduced in all 4I domains (involvement, integrity, interaction, introspection) and in their severity. Formal learning interventions (15 vs. 5) and informal learning plans (3 vs. 11) were significantly different in the pre- and post-Casper cohorts respectively (*p* < 0.05). This reduction in formal learning interventions was associated with a 96% reduction in costs f(rom hundreds to tens of thousands of dollars and a reduction in time for learning interventions (from years to months).

**Conclusions:**

Justifiable from multiple stakeholder perspectives, use of an SJT (Casper) improves a clinical performance measure (professionalism concerns) and permits the institution to redirect its limited resources (cost savings and time) to enhance institutional endeavors and improve learner well-being and quality of programs.

## Background

Admission into medical school and residency training programs is a high-stakes process as it affects the careers and well-being of the individuals who apply, care and health of the patients and communities, and the quality, costs and reputation of the institutions involved in medical education. Selections into medicine are aimed at identifying candidates with desirable attributes and deselecting those who are unlikely to be successful in that specialty or serve the society as physicians [1]. Further, there are many considerations that play a significant role in the complex process of selections. These include perspectives of different stakeholders that may not always be aligned [2, 3] and sometimes be in conflict. These include societal considerations that underlie the move towards a diverse workforce to meet culturally aligned healthcare needs of population and an equitable representation of societal segments in medicine. The applicants desire a process that is transparent, less expensive, maximizes their chances for selection into a specialty of their choice, and assesses those aspects over which they have some control. Institutions are motivated by their vision and mission including the social accountability mandate, differentiation, scope of influence, quality of education, selection of best possible candidates, reputation, operational efficiency, and resource utilization.

Selection processes into medical schools and postgraduate residency programs involve assessment of both cognitive abilities (CAs) and non-cognitive abilities (NCAs). The cognitive abilities (CAs) refer to general intelligence and learned knowledge [4] and the non-cognitive abilities (NCAs), sometimes referred to as socio-emotional skills [5], personal and professional characteristics [6], personal qualities [7], non-academic attributes [8], soft skills, or character skills are rather broad and include mindsets, attitudes, integrity, personality traits, learning strategies and social skills including communication and empathy. The CAs and NCAs are not mutually exclusive [9], are somewhat overlapping constructs [10] and both are required for effective practice of medicine [11]. The NCAs are considered essential for good physicians [12], better patient outcomes [13, 14], and underlie excellence by residents and physicians [15, 16]. 

Selection processes consider CAs and NCAs along with prior achievements, expression of interests and other factors in a somewhat structured and deliberate but quite variable manner, through a multitude of tools (e.g., interviews, computerized tests, personal letters, references, and others) aimed at identifying candidates with the highest likelihood of success. The importance of reliable tools [17] and assessment of wide-ranging attributes [18], is well-accepted. CAs are assessed using prior academic performance and tests of medical knowledge and weighted variably along with the assessment of NCAs in the processes to make final decisions [18]. 

The assessment of NCAs is considered worthwhile [19–21]. Indeed, a few studies have shown the relevance of personal characteristics to future success, including clinical performance and professionalism lapses [22] and overall competency [23]. Even more relevant is the demonstration of incremental validity of NCA assessment over and above academic assessment [22, 24]. Assessment of NCAs is highly variable and largely suboptimal [1], except when utilized through a validated process e.g., structured personal essays [25], multiple-mini interviews (MMIs) [26], or online situational judgment tests (SJTs) [27]. 

Computer-based assessment for sampling personal characteristics (Casper) is an online SJT that measures personal and professional qualities (computer-based video generated written response in an open-ended manner) in a contextualized manner and is devoid of medical knowledge assessment [28]. Contextualized assessment is essential since context is important for behavioral manifestations [29]. Casper has been used in selections in medical education settings [30, 31]. Casper has been generally used in undergraduate medical school admissions and only more recently for selections in postgraduate residency programs. It is used for assessment of NCAs e.g., receptivity to feedback empathy, teamwork, communication, collaboration, resilience, self-awareness, problem solving and ethics. It has good psychometric properties. Construct validity is ensured as the components of the test are based on the non-medical expert roles captured in various competency frameworks for medical education. The overall test generalizability and interrater reliability are high (G = 0.78–0.87) and (G = 0.81–0.92) respectively; and concurrent validity with MMI after correcting for dis-attenuation is 0.6, although the it varies based on MMI format [28]. Casper’s predictive validity for personal/professional components of national licensure examinations three-six years post-medical school admission has also been demonstrated (*r* = 0.3–0.5) [6]. Its discriminant validity is shown by a consistent absence or negative correlation with cognitive aspects of national licensure examinations [6]. 

However, the need for “a more solid empirical basis” [32] for the assessment of NCAs at the time of selections has been asserted from an incremental validity and utilitarian perspective. This includes demonstration of incremental validity (over and above assessment of academic performance) for predicting performance, especially clinical performance, and not just academic performance. The literature on predictive validity for performance in practice is extremely limited [33], with the work linking unprofessional behaviors to future adverse actions, being a rare example of how powerful such research can be. From the utilitarian perspective, there needs to be an immediate practical effect of using a selection instrument. For example, economic consequences of implementing selection tests are important considerations for medical institutions [34, 35]. There is a paucity of data linking Casper to future professionalism behavior. Further, we are not aware of any study that has addressed the impact of Casper on institutional resources and operations involved in remediations. Against the background of the need to uphold multiple stakeholder perspectives, the purpose of this study was to determine the impact of Casper in postgraduate residency selections (incorporated as a mandatory component of selections at our institution in 2017) on a clinical performance outcome measure - professional behavior, and the impact on institutional resources. This study adds to the literature by addressing these considerations and is one of the first forays into these areas and sets the stage for future research in this area.

## Methods

This study was deemed exempt by the University of Saskatchewan Ethics Review Board.

### Setting

Canadian residency selections are a two-iteration “match” process through the Canadian Resident Matching Service (CaRMS) and involve assessment of the applicants by the programs and the evaluation of the programs by the applicants to determine compatibility. Following rank order list generation, CaRMS algorithms find a match. Our institution is a participant in this national process. The reason to explore Casper was to assess all applicants for the NCAs in a standardized manner. The institution has no financial gains through this test offering.

Over a three-year period following a review of literature discussions were held with multiple stakeholders. This included the senior leadership of the College of Medicine, educational administrators of residency programs, townhalls with undergraduate students, presentations by the Casper team to the formal postgraduate education committee meetings and a formal voting process to adopt Casper. After this decision, a blueprinting exercise with the program directors to determine attributes considered necessary for success during residency and clinical practice. The findings were taken into consideration by Altus Assessments (now Acuity Insights) to determine if their current offering met these requirements or anything else needed to be added to their test menu. The first offering (2016) was voluntary (to conduct correlation studies with other selection methods) and results were not used in selection decisions. A small number of candidates (*n* = 56) took this test and there was concordance in the predictive ability of Casper scores for rank ordered lists generated by the traditional selection process. The test was then mandated for all programs commencing next year. The test results are used at our institution in the following manner. The raw and z scores (both institution-wide and program-specific), percentiles, and any narrative comments (highlighting any concerns) for all applicants are sent to the University and shared with the residency program selection committees. The programs must use Casper results in their deliberations in any one of the following three ways – either (a) for screening or (b) for quantitative part of their deliberations or (c) use it as a part of the overall qualitative discussion. Of the 25 programs, all programs use it for screening, 18 use it as a quantitative variable in their deliberations and 13 use it part of the overall discussion about the candidate (because overall assessment of the applicant involves interviews with SJT and BI components).

### Tool

The components of Casper for postgraduate medical education (PGME) residency selections at our institution include collaboration, communication, empathy, equity, ethics, motivation, problem-solving, professionalism, resilience and self-awareness, receptivity to feedback, judgement, and teamwork. The examinations are marked by independent fully trained assessors situated remotely.

### Participants

The study pool included residents selected each year as first year residents in direct entry programs over a three-year pre- Casper (2013–2015) and three-year post- Casper (2017–2019) time-period. Although follow-up data was available for many years (ranging from 3 to 9 years), only three-year post-selection data was used for each cohort as this was the minimum time of follow-up data for the 2019 cohort at the time of the study.

### Data collection and outcome measures

Data for this study was obtained from admission documents and education files of residents who were in difficulty (RID), defined as those who needed a learning intervention during their residency training. This study is based on secondary use of admissions data and information in the files of residents in difficulty, as the information was not primarily collected for this purpose and therefore required a review and subsequent exemption by the Ethics Board of the institution. RID files were compiled within the PGME office by the academic processes and policies coordinator and only anonymized and summarized information was conveyed to the project team.

We utilize a systematic tiered approach (addressing learners, teachers and system issues) reflecting the principles outlined in the graduated interventions for disruptive behavior model [36] and educational practices in different zones [37]. Criteria for learning interventions were reviewed subsequent to implementation. Changes to CBME policies spanned programmatic approach to assessments, resource allocation, modification of learning experiences including simulation to acquire competencies, and program evaluation. Changes to assessment involved a more comprehensive evaluation of all assessment data (including daily feedback that specifically required identification of professionalism and patient safety concerns and a more robust approach to assessment in clinical and academic settings). The criteria for R and P were reviewed and were not changed with the implementation of competency-based medical education (CBME); these remained same for pre- and post-Casper groups.

The educational interventions for RID could be either an enhanced learning plan (ELP), remediation ( R) or probation (P). The ELPs are informal and for those issues that do not meet the threshold for remediation or probation and are less intensive; these are usually 3 months in duration. The criteria for a formal learning intervention (R and P) are stringent reflecting the intensity of deficits, egregiousness of behavior and impact on the safety of the patients and learners. An ELP is a deliberately designed and structured learning plan intended to guide the resident towards successful attainment of specific competencies when it has been assessed that the deficiencies are mild and can be addressed with more focused attention. Remediation is a more rigorous intervention with clearly defined learning and assessment measures, and outcomes for residents experiencing considerable difficulties, according to our established criteria. Probation is the formal modification of the residency training to address specific identified weaknesses and where the extent of weaknesses is such where the resident’s ability to continue training is likely to be significantly compromised. R and P are usually six months in duration and can be as along as 12 months. The identification of areas for improvement / concerns are documented systematically through a formal programmatic assessment process and discussions and decisions by a resident assessment committee (prior to the adoption of competency-based medical education (CBME) and by the competence committee (after the implementation of CBME). The learning plans are developed at the program level involving the residency program committee utilizing standardized templates and these plans are discussed with the resident, faculty supervisors and mentors. These are then reviewed by the PGME office for adherence to policies and procedures, academic rigor and well-being and support for the resident. After formal approval these are implemented by the program. Regular monitoring and a review of the final report are done by the PGME office including approval of the actions based on the outcomes.

The following outcome measures were collected from resident files; (a) residents in difficulty (number of RID, frequency of different CanMEDS roles requiring interventions), (b) category of learning intervention (ELP, R, or P), (c) outcome of learning interventions (successful completion, adverse outcome including another learning intervention or termination, and appeal of the decision), and (d) costs and time associated with addressing RID (summary data provided by the PGME Resident Resource Office, Education Coordinator and the Finance Coordinator).

Professionalism concerns were considered using the 4I framework [38, 39], which originated in the undergraduate medical education setting and with minor changes has been shown to be relevant to the PGME settings [40]. The 4I framework articulates 30 specific unprofessional behaviors and maps these to four categories (involvement, integrity, interaction and introspection). The behavioral descriptors provide a guide to the assessors and educators on what to observe and document [38]. Based on another study [40], two descriptors were added to the Introspection category; these included absences related to perceived workload complaints (nervous exhaustion) and a nine-to-five mentality. Professionalism concerns were mapped onto the 4I domains and specific behavioral descriptors based upon the documentation in the program and the central PGME office files and the frequency of the specific behaviors recorded in the pre- and post-Casper cohort. The general categorization of professionalism concerns into mild, moderate and major was according to the descriptors from the Baylor College of Medicine [41]. 

### Analysis

Descriptive statistics was used for frequencies of quantitative data within groups e.g., number of residents, CanMEDS roles requiring intervention, category of learning interventions, and learner outcomes. Between group comparisons were performed using two-proportion z-tests on the number of learning interventions observed between the pre- and post-Casper groups, frequency of CanMEDS roles identified and the type of outcome.

## Results

The results are presented to reflect the two main questions of this study, a) association between Casper and professionalism behavior and the impact on institution (costs and time required for learning interventions).

### Number of residents

The number of first-year residents admitted into PGME pre- (2013–2015) and post (2017–2019) Casper were 361 and 343 respectively. Amongst these, the number of RID were similar (16 (4.4%) in the pre-Casper cohort and 15 (4.4%) in the post-Casper cohort.

### CanMEDS roles requiring attention

The medical expert domain was the most frequent role requiring attention in both the pre- and post- Casper cohorts. Professionalism concerns were identified in 75% of the pre- Casper cohort and 40% in the post- Casper group; a 35% reduction. The differences in the two groups across CanMEDS roles are shown in Table 1.

**Table 1:** Quantitative data on resident numbers, CanMEDS roles associated with residents in difficulty, learning interventions, learner outcomes and financial resources

Area	Pre-Casper	Post-Casper	Change	Comments; *p* value
CanMEDS roles requiring intervention				
Role	Pre-Casper	Post-Casper	Change	
Medical expert	16/16	12/15	20% ↓	ns
Professional	12/16	6/15	35% ↓	*P* < 0.05
Collaborator	5/16	1/15	24% ↓	ns
Leader/Manager	6/16	3/15	18% ↓	ns
Scholar	5/16	2/15	18% ↓	ns
Health Advocate	1/16	1/15	Similar (1%↑)	ns
Communicator	8/16	8/15	Similar (3%↑)	ns
Learning Interventions				
Enhanced learning plans	3	11		*P* < 0.05
Remediations/Probations	15	5		*P* < 0.05
Total number of interventions*	18	16		ns
Unfavorable learner outcomes				
Terminations and Appeals**	Terminations = 3 Appeals = 3	Terminations = 0 Appeals = 0		*P* < 0.05
Financial Resources required				
Costs (excluding legal costs)	Hundreds of thousands	Tens of thousands	96% ↓	Excludes legal costs

↓ decrease; ↑ increase

*The number of learning interventions is higher than the number of learners in difficulty as some learners required more than one intervention

**The decisions and the initial appeals occurred within the three year follow-up period, however, the final decisions after all appeals and legal processes were exhausted took longer than three years and were outside of the study period

### Professionalism concerns

Using the 4I framework the identified themes/ categories and specific behavioral descriptors of professionalism concerns are shown in Table 2. In addition to the overall reduction in the number of residents with professionalism concerns (35% reduction), there was a decrease in the number of professionalism concerns in all four domains; 17% each in the involvement, interaction, and introspection domains, and a 25% reduction in the integrity domain. Further the “severity” of the concerns in the post- Casper group was considered to be mild to moderate and not major.

**Table 2:** Change in the specific categories and behavioral descriptors of unprofessional behavior after implementing Casper. The numbers associated with behavioral descriptors are more than the number of residents exhibiting unprofessional behavior as more than one behavior was exhibited by some residents. The 4I framework and the original tabular format of unprofessional behaviors is used with permission from Dr. M van der Vossen. As described in the Methods section, two behavioral descriptors were added to the Introspection category (nervous exhaustion and none-to-five mentality) and no residents exhibited these behaviors in either cohort

**INVOLVEMENT (Failure to engage)**			**INTEGRITY (Dishonest behaviors)**		
	**Pre-**	**Post-**		**Pre-**	**Post-**
Absent or late for assigned activities	6/12	3/6	Cheating in exams	0/12	0/6
Not meeting deadlines	9/12	5/6	Lying	2/12	0/6
Poor initiative	4/12	3/6	Plagiarism	0/12	0/6
General disorganization	5/12	5/6	Data fabrication	2/12	0/6
Cutting corners	4/12	3/6	Data falsification	3/12	0/6
Poor teamwork	3/12	2/6	Misrepresentation	2/12	0/6
Language difficulties	0/12	0/6	Acting without required consent	3/12	0/6
			Not obeying rules and regulations	9/12	3/6
Number of residents			Number of residents		
Pre-Casper (12/12; 100%) à post-Casper (5/6; 83%); 17% reduction			Pre-Casper (9/12; 75%) à post-Casper (3/6; 50%); 25% reduction		
**INTERACTION (Disrespectful behavior)**			**INTROSPECTION (Poor self-awareness)**		
	**Pre-**	**Post-**		**Pre-**	**Post-**
Poor verbal/non-verbal communication	8/12	3/6	Avoiding feedback	5/12	2/6
Inappropriate use of social media	0/12	0/6	Lacking insight in own behavior	10/12	4/6
Inappropriate clothing	0/12	0/6	Not sensitive to other person’s needs	3/12	1/6
Disruptive behavior in teaching sessions	2/12	0/6	Blaming external factors rather than own inadequacies	5/12	3/6
Privacy and confidentiality violations	1/12	1/6	Not accepting feedback	10/12	3/6
Bullying	1/12	0/6	Resisting change	7/12	1/6
Discrimination	0/12	0/6	Not aware of limitations	7/12	4/6
Sexual harassment	0/12	0/6			
Number of residents			Number of residents		
Pre-Casper (8/12; 67%) à post-Casper (3/6; 50%): 17% reduction			Pre-Casper (12/12; 100%) à post-Casper (5/6; 83%): 17% reduction		

### Changes in the type of learning interventions required

Formal learning interventions (15 vs. 5) and informal learning plans (3 vs. 11) were significantly different in the pre- and post- Casper cohorts respectively (*p* < 0.05).

### Outcomes of intervention

In the pre- Casper cohort, more than one formal intervention (remediation or conversion of remediation to probation or ELP followed by remediation or probation) occurred in 3 cases; while in the post- Casper cohort, none of the residents required another learning intervention.There were no terminations in the post- Casper cohort compared to three in the pre- Casper group (two dismissals and one leave of absence and resignation).

### Financial impact on the publicly funded PGME

The costs associated with salary for additional training, preceptor remunerations, additional assessments to tailor interventions, logistics (vacations, leaves, travel), and resident resource office support were reduced by 96% (from hundreds of thousands to tens of thousands of dollars). Addition of legal costs to this equation reduced it by approximately 99%. The savings were redirected to enhance residency education; specifically, improve the quality of the programs (infrastructure support – technical, physical, materials, simulation, academic programming – a wider access to courses and conferences), and resident well-being. Some of the funds were utilized to support institutional endeavors e.g., the continuing implementation of CBME.

### Time required for learning interventions

The time spent on all learning interventions was measured in years for the pre-Casper cohort and in months for the post-Casper cohort. The “time” included actual faculty time during learning interventions and time for development of plans, and processes and administrative oversight. It did not include time for appeals and legal processes.

## Discussion

The introduction of Casper as a mandatory requirement for entry into postgraduate residency programs led to a reduction in the number of professionalism concerns (and various domains and specific behavioral descriptors as outlined in van der Vossen framework), reduction in the number of formal learning interventions (remediations and probations), correction of identified professionalism deficiencies with less intense enhanced learning plans and a marked reduction in the use of institutional resources (time required and institutional costs).

The two most important contributions in the assessment of personal characteristics have been incorporation of behavioral and situational judgment aspects in various methods including interviews [26], personal essays [25], online tests [6, 42], and selection centres [43]. Predicting future performance through outcome variable(s) is often used to justify the use of a selection method, tool or an approach. Professionalism concern(s) are a useful clinical performance outcome measure as a decrease in professionalism concerns is relevant to the practice of medicine and its role in professional identity formation [44]. Further, there is a linkage between unprofessional behaviors during medical education and subsequent adverse actions by the regulatory bodies [33]. Using an SJT derived test is useful to assess NCAs, as SJTs are predictive of both performance at later stages of selection process itself, e.g., performance in selection centres [45] and predicting rank order list in a manner similar to traditional faculty assessments of applicants [46], and performance upon entry into clinical practice [47]. 

One confounding variable in our study was the early stages of CBME implementation at our institution in the same timeframe. It is possible that CBME had an effect, especially for those residents who in the post-Casper cohort were in programs that were in the early stages of CBME implementation, however, the effect would have been in the opposite direction, i.e., more concerns are likely to have been identified due to enhanced supervision and increased rigor (frequency and deliberate focus including on professionalism and patient safety) of feedback. Almost all residents in difficulty in the post- Casper cohort demonstrated willingness to take responsibility and address the issues and ranked high on both reflectivity and adaptability; [48] this, along with less severe concerns – mild and moderate only [41] - contributed to the development of enhanced learning plans as opposed to remediations and probations.

Selections into medical school and later into residency programs need to balance the needs of the applicants, institutions, and the society at large. The process must be fair and transparent for the applicants and the institutions. The applicants’ perceptions of selection practices are important [49]; if perceived to be unfair, many excellent candidates may choose not to apply. Since our institution was the first to mandate this test in Canada in PGME admissions, there was a 3% decrease in the number of applicants in the pilot year (2016), but from the next year onwards it has been consistently increasing. Our goal was for all applicants to have an opportunity to be assessed for personal characteristics and not just those who have been screened by either academic records or personal letters that are limited by low reliability coefficients [50] or reference letters that have significant interpretive variability [51]. Offering an opportunity to all applicants to be assessed for the abilities essential for the practice of medicine “job” is in keeping with an organizational justice perspective [52]. This objective assessment is even more important if the interviews at the program level are not structured, and the interviewers have not been trained who then engage in assessing NCAs in a variable manner, especially in view of the low reliability of the interviews [53]. Finally, the test itself should not be subject to improvements due to coaching; as an SJT, coaching (for performing on the test) does not cause any more effect [54] that it does on cognitive tests [55].

An additional consideration is the cost of the test to the applicants and the institutions. There is indeed a cost to write another examination, however it is probably the cheapest (cost approximately 60 CAD) exam to write (compared to other exams). Further, from an institutional perspective SJTs are cost effective [56]. The institutions need to be prudent in the use and allocation of resources (especially when using public money), operationally efficient to achieve intended outcomes. The cost of residency training is high, estimated to be approximately $ 100,000 / year / resident (approximation from our data). The real and hidden costs of remediation and not remediating a resident who needs it are high [57], and if professionalism is a reason then these tend to be even higher. The costs of unprofessional behavior in the healthcare system are also very high [58] and addressing these issues at selection and during residency is worthwhile. However, the resources that are not required for these corrections can be and have been utilized to improve the overall quality of the educational programs, large-scale endeavors to improve well-being and by investing in efforts to make good students even better.

There are additional tangible and intangible costs, and among others include time (additional teaching, supervision, administrative processes and oversight, and appeals), and morale. There are other considerations for institutions as well, including the risk to institutional reputation, and the political impact of going alone. There was considerable opposition at the beginning and the necessary strong leadership support was provided by the Dean and Vice Dean of our institution to sustain the efforts to implement this endeavor. Following our lead, many other institutions in postgraduate settings have adopted Casper.

Working towards equitable representation in medicine is one consideration when thinking of societal implications. Inclusion of non-academic data (to supplement academic data) is even more important for disadvantaged and minority applicants [59]. SJTs have been shown to have a relatively low adverse impact on minority applicants and have the potential to reduce the disadvantage of the lower socio economic status but not across ethnicity [27]. Further SJTs are generally perceived to be fair by the applicants [56]. The evidence for bias of Casper across societal segments is conflicting and evolving. On the one hand there is no evidence of bias for age, gender and aboriginal status for Casper [60] and the differences between groups across intersectionality variables are smaller for Casper and MMI (compared to academic assessments) and a higher weighting of Casper has been proposed to increase diversity across race, gender and ethnicity [61]. On the other hand, studies have identified Casper percentiles to be significantly different for gender [62], race [62], language use (bilingual vs. English only or English as a second language) [63], and socio-economic status [63]. In general, between group differences between groups across societal segments are smaller in the SJTs that what is typically observed with other academic evaluations [8, 61, 63]. Importance of group differences highlights the need to consider adjusting testing to minimize those differences. For example, to reduce bias in Casper, altering response format from typed to a video response, was recently shown to substantially lower group differences across race, socioeconomic status, language use and rural vs. non-rural background (assessed by determining effect size using Cohen’s d) [64]. 

## Conclusions

Simultaneously addressing multiple stakeholder perspectives is a balancing act between meeting applicant, societal and institutional needs, and accountability to our funders for the most prudent use of money for the highest quality education. Our study has added to the literature that online assessment of personal characteristics in a standardized manner resulted in improvements in an important outcome measure – the incidence and severity of professionalism concerns were reduced, required informal ELPs to address the deficiencies and were associated with a marked reduction in the institutional resources (financial and time) required. The limitations of this study include a relatively small sample size, limited follow-up (three years for each of the six cohorts), a single institutional perspective and absence of details on systemic factors affecting professionalism behaviors, and any unaccounted pre-existing differences between cohorts.

The implications of our study include suggestions for a process for adoption of a new test(s) for selections in residency programs, integration of a validated and objective tool for assessment of NCAs in a comprehensive approach for selections, training of reviewers to consider its use in applicant deliberations, and the use of institutional data to determine the impact and decision to continue the use. Future research aimed at exploring resident outcome analysis of longitudinal data over a longer time-period focused on predictive validities of selection measures is needed. It will also be helpful to include a wider array of outcome measures of both academic and clinical performance to more fully explore the impact of incorporating SJTs (e.g., Casper) in the selection processes in PGME. There are significant advantages from postgraduate programs in terms of collection, analysis, interpretation, and dissemination of postgraduate data compared to undergraduate counterparts. These advantages include greater trainee homogeneity and thus easier interpretation; greater degree of longitudinal data available for analysis, as at this point it includes the complete undergraduate data set in addition to at least part of the postgraduate; less lead time to performance in practice, thus shortening time to obtaining interpretable and actionable results; and fewer confounding factors when seeking correlation in post-training outcome measures of performance in practice.

## Data Availability

The datasets used and/or analysed during this study are available from the corresponding author on reasonable request.

## Abbreviations

Casper: Computerized assessment of personal attributes
CAs: Cognitive abilities
CanMEDS: Canadian Medical Education Directives for Specialists
CaRMS: Canadian resident matching service
CBME: Competency-based medical education
ELP: Enhanced learning plan
MMI: Multiple-mini interviews
NCAs: Non-cognitive abilities
P: Probation
PGME: Postgraduate Medical Education
R: Remediation
SJT: Situational judgement test
